# Is Immunohistochemical Galectin-3 Expression Associated with the Epithelial–Mesenchymal Transition in High- and Low-Grade Invasive Urothelial Carcinomas of the Bladder? †

**DOI:** 10.3390/diagnostics14202270

**Published:** 2024-10-12

**Authors:** Merve Cin, Ayşenur Akyıldız İğdem, Sibel Bektaş, Özgecan Gündoğar, Selçuk Cin, Neslihan Komut, Buğra Çetin

**Affiliations:** 1 Department of Pathology, Istanbul Training and Research Hospital, University of Health Sciences, 34098 Istanbul, Turkey; 2 Nishantashi Pathology Group Laboratory, 34365 Istanbul, Turkey; aysenurigdem@gmail.com; 3 Department of Pathology, Gaziosmanspasa Education and Research Hospital, University of Health Sciences, 34255 Istanbul, Turkey; sibel_bektas@yahoo.com (S.B.); ozgecankarahan@hotmail.com (Ö.G.); 4 Department of Pathology, Bagcilar Training and Research Hospital, University of Health Sciences, 34200 Istanbul, Turkey; selc2049@hotmail.com; 5 Department of Pathology, Tekirdag Dr. Ismail Fehmi Cumalioglu City Hospital, 59030 Tekirdag, Turkey; neslihankomut@gmail.com; 6 Department of Urology, Bahcelievler Medicalpark Hospital, Altinbas University, 34180 Istanbul, Turkey; cetinbugra@yahoo.com

**Keywords:** bladder cancer, galectin-3, epithelial–mesenchymal transition (EMT), E-cadherin, vimentin

## Abstract

**Background/Objectives**: Bladder cancer, predominantly urothelial carcinoma, is an important malignancy of the urinary system. Despite the same histologic grade and stage, some patients seem to have a worse prognosis. In this context, the epithelial–mesenchymal transition (EMT), characterized by the loss of E-cadherin and gain of vimentin expression, is an important process in tumor progression. Galectin-3, a lactose-binding protein involved in various cellular processes, has been associated with increased tumor cell migration, invasion, and treatment resistance. **Methods:** In this study, 223 bladder cancer cases were examined, and E-cadherin, vimentin, and galectin-3 expression was evaluated by immunohistochemical staining in tumor budding areas and invasive components. These markers were also correlated with clinicopathological parameters, including tumor grade and stage. **Results:** The results indicated a significant decrease in E-cadherin expression and an increase in vimentin staining in higher-grade and higher-stage tumors, supporting EMT involvement. Galectin-3 expression was notably higher in T1 high-grade tumors but decreased in T2 stage tumors. Despite this, no significant correlation was found between galectin-3 and E-cadherin or vimentin, suggesting a complex role of galectin-3 in EMT. **Conclusions:** High galectin-3 expression in T1 high-grade tumors highlights its potential role in early tumor progression and as a therapeutic target. However, the decrease in its expression in advanced stages underscores the need for further research to understand its multifaceted involvement in bladder cancer. These findings suggest that while galectin-3 may contribute to the EMT and early tumor progression, its exact role and potential as a therapeutic target require more detailed investigation.

## 1. Introduction

Bladder cancer represents the most prevalent malignancy of the urinary tract, with an anticipated 83,190 new cases in the United States for 2024. It ranks as the fourth most common cancer among men and is the eighth leading cause of cancer-related mortality expected in the same year [1]. Urothelial carcinoma constitutes over 90% of all bladder cancer cases. Of these, approximately 75% are classified as either non-invasive or invasive to the lamina propria, while roughly 20% exhibit muscle invasion [2].

Despite patients being at the same stage, histologic grade, and histologic type, variations in prognosis have been observed. Recent research has highlighted the concept of tumor budding as a significant factor in many malignancies, particularly colorectal carcinomas [3,4]. Tumor budding is considered a morphological manifestation of the epithelial–mesenchymal transition (EMT). During this process, tumor cells adopt mesenchymal characteristics while diminishing or losing their epithelial properties. Consequently, tumor cells that acquire motility are associated with more advanced disease stages, increased lymphovascular invasion, and a higher propensity for metastasis. One of the principal alterations observed during the epithelial–mesenchymal transition (EMT) involves E-cadherin, a protein crucial for cell–cell adhesion. In certain tumor cells of epithelial origin, E-cadherin expression is reduced or entirely lost. Conversely, vimentin, an intermediate filament characteristic of mesenchymal cells, begins to be expressed in these tumor cells. This shift in expression patterns is recognized as a key morphological and immunohistochemical marker of the EMT process [5,6,7,8,9].

Galectins constitute a family of lactose-binding proteins that exhibit a notable affinity for β-galactoside residues and display substantial sequence homology within their carbohydrate-binding domains [10]. Currently, 15 distinct members of the galectin family have been identified [11]. Galectin-3 is implicated in a diverse array of cellular compartments, including the nucleus, cytoplasm, and cell membrane, and it is also associated with the extracellular matrix. Due to its multiple functions and various locations, it is differentially expressed in both normal and neoplastic cells [10,11]. Galectin-3 is recognized for its role in various cellular processes, including adhesion to the extracellular matrix in tumor cells, facilitation of invasion and migration, promotion of angiogenesis in both primary and metastatic tumor sites, inhibition of apoptosis, and modulation of T cell activation [12,13]. Studies in breast cancer have demonstrated that cells overexpressing galectin-3 exhibit increased migration, whereas downregulation of galectin-3 expression is associated with reduced tumor growth and diminished migratory capacity [12]. Galectin-3 plays a crucial role in enhancing the invasive capabilities of tumor cells by promoting the disruption of the basement membrane through the upregulation of matrix metalloproteinase activity [12]. Additionally, the interaction between integrin and galectin-3 has been shown to facilitate cell migration by diminishing the binding of integrin to collagen in the extracellular matrix [14]. These observations underscore the involvement of galectin-3 in the epithelial-to-mesenchymal transition (EMT). Given its contribution to treatment resistance and the impact of therapeutic strategies aimed at inhibiting galectin-3 function, it is imperative to consider galectin-3 in treatment management [12,15,16,17].

Given the complex roles of galectin-3, particularly in the epithelial-to-mesenchymal transition (EMT), and the variability in its expression reported in bladder urothelial carcinomas, this study seeks to elucidate several key aspects. Specifically, we aim to analyze the EMT in bladder urothelial carcinomas using E-cadherin and vimentin as markers. Additionally, we evaluate galectin-3 expression in invasive regions with a focus on tumor budding areas and investigate its association with critical prognostic parameters such as tumor stage and histological grade. Furthermore, we intend to explore potential correlations between E-cadherin and vimentin, which are used for immunohistochemical detection of the EMT, and galectin-3.

## 2. Materials and Methods

### 2.1. Definitions of the Study Group and Clinicopathologic Parameters

This study included TUR (transurethral resection) specimens from 223 cases diagnosed at the Department of Medical Pathology, University of Health Sciences Gaziosmanpasa Training and Research Hospital, over a period of 9 years. This study started with 379 bladder TUR materials containing invasive urothelial carcinoma. Ninety-two cases were excluded because they did not contain a muscle layer. Thirty-four patients had more than one TUR material and a total of ninety-eight TUR materials containing a muscle layer were available for these thirty-four patients. If there was more than one TUR material from the same patient, the sample containing the most invasive tumor area was preferred. Only one specimen from each patient was included in this study. Thus, 64 recurrent TUR materials were excluded from this study. Data on the 223 cases were extracted from pathology reports and the hospital’s information recording system.

### 2.2. Histomorphological Evaluation

All hematoxylin and eosin (H&E)-stained preparations from the TUR materials of the 223 cases were retrieved from the archive and re-evaluated to assess tumor grade, lymphovascular invasion, presence of concomitant differentiation (such as squamous, micropapillary, glandular, or sarcomatoid), depth of invasion, and the presence of carcinoma in situ (CIS). Tumor grading followed the criteria outlined in the WHO classification for urinary and male genital tumors, 5th edition, while tumor staging adhered to the AJCC 8th edition guidelines. Cases with invasion into the lamina propria were classified as T1 stage, and cases with invasion into the muscularis propria were classified as T2 stage [2,18]. The cases were subsequently categorized into three groups: T1 low grade (T1LG), T1 high grade (T1HG), and T2 high grade (T2HG).

### 2.3. Immunohistochemical Evaluation

The slides exhibiting the most invasive tumor area and the highest degree of tumor budding were selected from each TUR specimen, and sections were prepared from paraffin blocks onto positively charged slides with a thickness of 3–4 microns. The immunohistochemical study was performed using a Ventana Benchmark XT automated stainer (Ventana Medical System, Inc., Tucson, AZ, USA) utilizing the following antibodies: E-cadherin (Thermo Fisher Scientific, Waltham, MA, USA Thermo Scientific, EP700Y/Monoclonal, dilution: 1:50), vimentin (Novocastra, Leica Biosystems, Newcastle Upon Tyne, UK, V9/Monoclonal, dilution: 1:800), and galectin-3 (CellMarque/SigmaAldrich, St. Louis, MO, USA, 9C4/Monoclonal, dilution: 1:25). The immunohistochemistry results for E-cadherin, vimentin, and galectin-3 were assessed according to the evaluation scale established by Raspollini et al. [19]. Staining intensity score (SIS) and staining extensity score (SES) were obtained for each marker (Table 1 and Table 2).

### 2.4. Statistical Method

Analyses were conducted using the NCSS 10 software program (2015, Kaysville, UT, USA). The age variable was assessed using a one-way ANOVA to compare differences between groups. Nominal variables were analyzed using chi-squared tests, Yates-corrected chi-squared tests, and Fisher’s exact probability tests. The relationship between variables was evaluated using the Spearman correlation test. Agreement between methods was determined using the Kappa statistic. A significance level of *p* < 0.05, with a bidirectional approach, was considered for all statistical tests.

## 3. Results

### 3.1. Association between Clinicopathologic Parameters and E-Cadherin, Vimentin, and Galectin-3

The distribution of clinicopathological findings across the T1LG, T1HG, and T2HG groups is summarized in Table 3. The distribution of cases with divergent differentiation was as follows. Thirty-six cases showed squamous differentiation, five cases showed glandular differentiation, five cases showed micropapillary differentiation, four cases showed sarcomatoid differentiation, and nine cases showed differentiation in more than two histologic types. Squamous differentiation was predominant in cases with more than two histologic types of differentiation.

E-cadherin (*p* < 0.001) and vimentin (*p* < 0.05) exhibited statistically significant positive correlations with increased SIS, SES, and the presence of lymphovascular invasion. In contrast, no statistically significant correlation was observed between galectin-3 SIS, SES, and its expression with the presence of lymphovascular invasion. The incidence of CIS was significantly higher with increasing histological grade (*p* = 0.009), with the T2HG group showing the highest rate of CIS, which was statistically significant. However, no correlation was found between tumor stage and the incidence of CIS. Additionally, there was no statistically significant correlation between the expression of E-cadherin, vimentin, and galectin-3 with the presence of CIS and concomitant differentiation.

**Figure 1:**
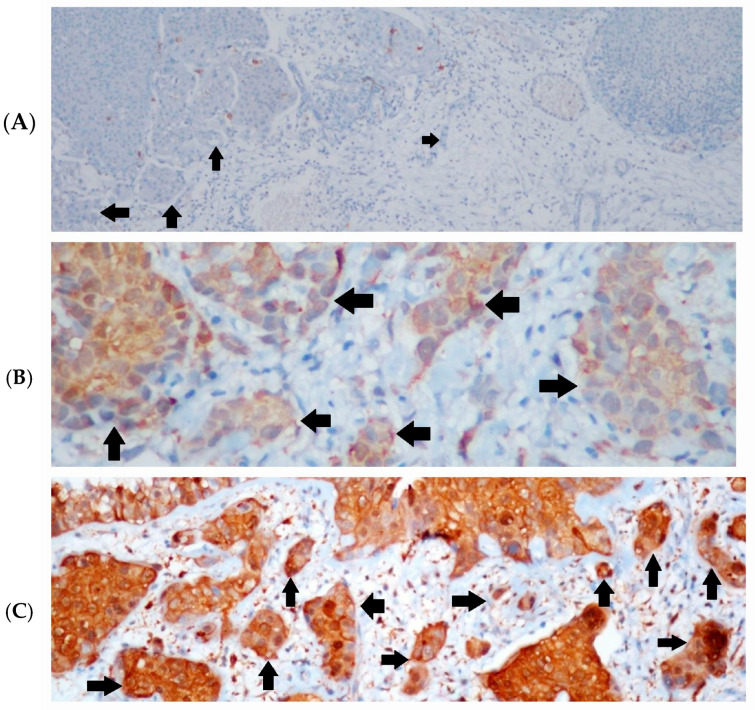
Immunohistochemical (IHC) staining intensities of galectin-3. (**A**) Invasive tumor islands without galectin-3 expression in low-grade urothelial carcinoma (black arrows), IHC × 100; (**B**) weak galectin-3 expression in invasive tumor area in high-grade urothelial carcinoma (black arrows), IHC × 400, (**C**) strong galectin-3 expression in invasive area with tumor budding in high-grade urothelial carcinoma (black arrows), IHC × 200.

In high-grade tumors, galectin-3 expression was observed in 67.6% of the T1-stage group and 45.7% of the T2-stage group. Galectin-3 staining was detected in 54% of low-grade cases, though it was predominantly weak (Figure 1A–C).

**Figure 2:**
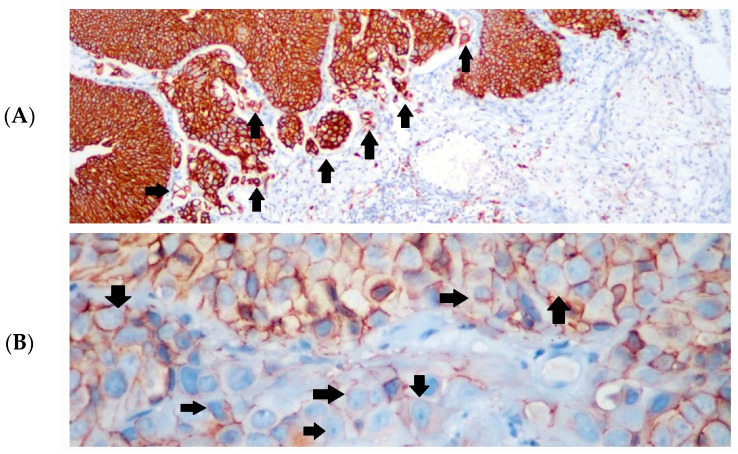

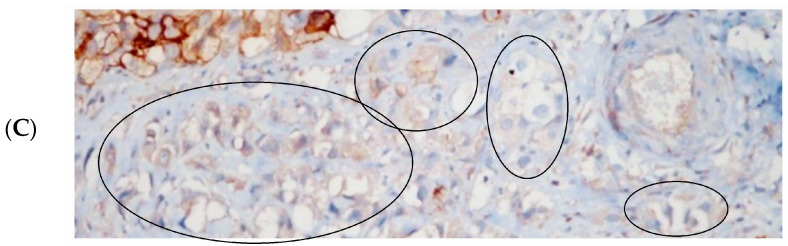
Immunohistochemical (IHC) staining intensities of E-cadherin. (**A**) Strong membranous E-cadherin expression in low-grade urothelial carcinoma (black arrows), IHC × 100; (**B**) weak membranous and cytoplasmic E-cadherin expression in invasive tumor cells in high-grade urothelial carcinoma (some of them are marked with black arrows), IHC × 400; (**C**) loss of E-cadherin expression in the invasive component of high-grade urothelial carcinoma (circles), IHC × 200.

A loss of membranous staining or the presence of cytoplasmic staining with E-cadherin was noted in 61% of cases in the high-grade group. In 20% of low-grade cases, cytoplasmic staining with E-cadherin was observed without a corresponding loss of membranous staining (Figure 2A–C).

**Figure 3:**
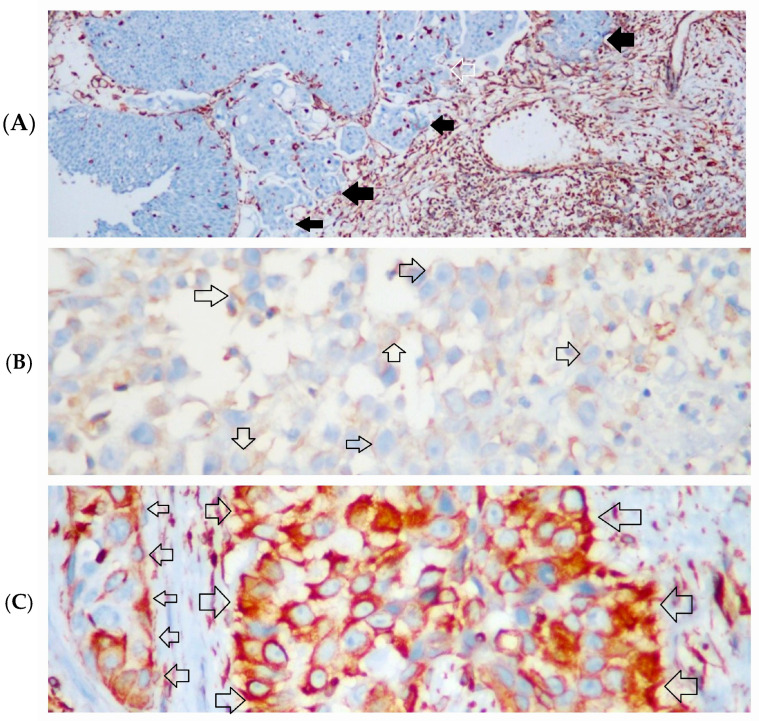
Immunohistochemical (IHC) staining intensities of vimentin. (**A**) Tumor cells without vimentin expression in low-grade urothelial carcinoma (black arrows). Stromal cells and inflammatory cells in the lower-right part are positive for vimentin, IHC × 100; (**B**) weak vimentin expression in invasive tumor cells in high-grade urothelial carcinoma (arrows with black borders), IHC × 400; (**C**) strong vimentin expression in invasive tumor islands in high-grade urothelial carcinoma (arrows with black borders), IHC × 400.

Vimentin staining was identified in 22% of high-grade tumors, whereas none of the low-grade tumors exhibited staining. Within our series, vimentin expression was detected in 14% of T1-stage cases and 29% of T2-stage cases (Figure 3A–C).

When the SIS and SES of galectin-3 in high-grade tumors were evaluated in relation to stage, a statistically significant correlation was observed (*p* < 0.001, *p* = 0.002, respectively). It was noted that the percentage and intensity of galectin-3 expression tended to decrease as the stage advanced. Furthermore, when comparing the galectin-3 staining patterns in low-grade and high-grade T1 stage cases with respect to grade, statistically significant results were found, with SIS and SES increasing as the grade advanced (*p* = 0.021 and *p* = 0.019, respectively). Consequently, the highest immune reaction of galectin-3 was observed in the T1 high-grade (T1HG) group. Additionally, the comparison of E-cadherin SIS and SES between the groups revealed a statistically significant loss of E-cadherin expression with increasing stage or histologic grade (*p* < 0.001). Although a statistically significant correlation was not detected when evaluating the intensity of vimentin staining in high-grade tumors across different stages (*p* = 0.082), there was an observable trend suggesting an increase in vimentin expression with advancing stage. However, when the percentage of vimentin staining in high-grade tumors was analyzed by stage, a statistically significant relationship was identified, indicating that the percentage of staining increased with progression of the stage (*p* = 0.008). The T2 high-grade (T2HG) group exhibited the highest levels of vimentin staining intensity and extensity (Table 4 and Table 5).

### 3.2. The Relationship between E-Cadherin, Vimentin, and Galectin-3

When examining the correlation between vimentin and E-cadherin, statistically significant correlations were observed for both the SIS (*p* < 0.001) and the SES (*p* < 0.001). This indicates a correlation between the loss of E-cadherin expression and positive vimentin expression. In contrast, no significant correlation was found between galectin-3 and E-cadherin for either SIS (*p* = 0.098) or SES (*p* = 0.35). Additionally, no significant correlation was detected between galectin-3 and vimentin for either SIS (*p* = 0.77) or SES (*p* = 0.61).

## 4. Discussion

The clinical stage and histological grade of the tumor are the most significant independent prognostic factors in bladder cancer, as demonstrated by various studies [2]. However, despite having the same stage, histological grade, and histological type, some cases exhibit more aggressive behavior, including frequent recurrences, early metastasis, and shorter survival times. This has led to the consideration that additional histopathological factors in the primary tumor may influence this process. Tumor budding and the epithelial–mesenchymal transition (EMT) are among these factors and have been studied extensively to predict prognostic outcomes in various epithelial tumors, including colon, cervix, and prostate cancers and bladder urothelial carcinoma [20,21,22,23,24,25]. Histomorphologically, tumor budding is characterized by tumor cells infiltrating the stroma individually or in small clusters of fewer than five cells, a feature identified as an important prognostic factor in colorectal carcinomas [4,24]. During the EMT, epithelial cells undergo a loss of E-cadherin expression, which is an epithelial marker, and transition to a mesenchymal phenotype, accompanied by the expression of markers like vimentin and N-cadherin, which are not typically present in epithelial cells [5,6,7,8]. Additionally, the involvement of other molecules in this process has been explored. Galectin-3’s role in EMT is evident due to its ability to enhance tumor cell migration and proliferation, contribute to metalloprotease activity in the extracellular matrix, and alter integrin–collagen interactions [12,14,26]. In this study, EMT was assessed immunohistochemically through changes in E-cadherin and vimentin expression in invasive tumor regions, and the expression of galectin-3 was investigated in these areas.

Numerous studies in the literature concerning urothelial carcinoma of the bladder and other organ tumors have linked the loss of membranous or cytoplasmic E-cadherin expression with poor prognosis [19,27,28,29]. However, Zhao and Köksal reported that a decrease or loss of E-cadherin immunoexpression was not associated with recurrence or rapid progression in bladder urothelial carcinomas [30,31]. In our study, E-cadherin expression was observed to decrease with T2 stage, high grade, and the presence of lymphovascular invasion—factors also associated with poor prognosis. Regarding vimentin staining, the expression of vimentin in bladder urothelial carcinomas was significantly associated with adverse prognostic indicators such as progression and recurrence [30]. Nonetheless, some studies present an opposing perspective. Singh et al. reported a 25% rate of vimentin expression in low-grade urothelial carcinoma; Baumgart et al. reported 6%, and Zhao et al. reported 36% [23,27,30]. Similarly, Paliwal et al. found this rate to be 0%, which aligns with our findings [32]. In high-grade urothelial carcinoma, reports in the literature on vimentin staining rates vary widely, ranging from 16% to 63%. In our study, vimentin staining was observed in 22% of high-grade tumors. According to the literature, vimentin immunoreactivity ranges from 7% to 36% in T1-stage tumors and 20% to 53% in T2-stage tumors [23,27,30,32]. In our series, vimentin expression was found in 14% of T1-stage tumors and 29% of T2-stage tumors. Baumgart et al., in a study of 874 cases, and Zhao et al., in a study of 121 cases, reported a statistically significant relationship of stage and histologic grade with vimentin expression [27,30]. Consistent with these findings, our study also revealed a statistically significant correlation between decreased E-cadherin expression, increased stage and histologic grade, and heightened vimentin reaction.

Canesin et al. reported that increased galectin-3 staining in bladder urothelial carcinomas was associated with progressive disease and reduced survival, while Kramer et al. found that decreased galectin-3 expression was linked to a higher frequency of recurrence in non-invasive urothelial carcinomas [33,34]. Conversely, two other studies did not find a significant association between galectin-3 and disease-free survival or life expectancy [35,36]. Regarding urothelial carcinoma grade, some studies have reported that high-grade tumors exhibit more galectin-3 expression compared to low-grade tumors [33,37]. However, Kramer et al. noted that low-grade tumors showed more galectin-3 immunoreactivity than high-grade tumors [34]. Two additional studies found no correlation between histologic grade and galectin-3 expression [35,38]. In our study, high-grade tumors at the T1 stage exhibited significantly greater galectin-3 reactivity than low-grade tumors. Furthermore, Gendy et al. found no significant correlation between Ta- and T1-stage tumors in terms of galectin-3 staining, while a significant positive correlation was observed between T1- and T2-stage tumors [37]. In the study by Kramer et al., which included 162 cases, and the study by Al-Maghrabi et al., with 128 cases, no correlation was found between tumor stage and galectin-3 expression [34,38]. Conversely, Canesin et al. and Langbein et al. reported a positive correlation between galectin-3 and both T1- and T2-stage tumors, noting that galectin-3 expression increased with advancing clinical stage [33,35]. In our study, galectin-3 expression was detected in 67.6% of T1 stage tumors and 45.7% of T2 stage tumors among high-grade cases. Research on galectin-3 in bladder urothelial carcinomas is limited, with discrepancies in sample sizes, antibody clones, and application methods contributing to seemingly contradictory findings. In our study, galectin-3 immunoreactivity was predominantly observed in the T1 high-grade (T1HG) group, with staining rates decreasing as the stage progressed. A key distinction in our study is its focus on the relationship between the EMT and galectin-3; thus, staining was assessed primarily in invasive tumor areas with pronounced tumor budding, while expressions in superficial non-invasive tumor regions were excluded from scoring.

Galectin-3 has been demonstrated to modulate resistance to EGFR inhibitors, regulate tyrosine kinase receptors, inhibit the apoptosis mechanisms of chemotherapeutic agents, and contribute to chemotherapeutic resistance through these pathways [39,40,41,42]. A preclinical study has reported successful outcomes with antibody therapy targeting galectin-3 in pancreatic cancers via EGFR, indicating that galectin-3 blockade reduced tumor cell migration, adhesion, and invasion [43]. In our data, galectin-3 expression was found to be highest in T1HG tumors. Studies have shown that cancer cells expressing galectin-3 exhibit stem cell-like behavior, can escape apoptosis, and have features such as T cell inactivation [12]. In this context, it can be predicted that the T1HG cases in our study may show rapid progression and have a poor prognosis, considering the high galectin-3 expression. We also think that these cases expressing galectin-3 may be followed up at more frequent intervals and clinicians should be vigilant in terms of treatment resistance.

In the study by Al-Maghrabi et al., no significant statistical relationship was found regarding galectin-3 expression in urothelial carcinoma cases with or without squamous differentiation [38]. In our study, we examined the relationship between concomitant differentiation (such as squamous and glandular differentiation) and E-cadherin, vimentin, and galectin-3 immunoreactivity in papillary urothelial carcinoma. Although a significant difference was observed between the groups in terms of differentiation (*p* < 0.001), no significant correlation was found between E-cadherin, vimentin, and galectin-3 expression and the presence or absence of concomitant differentiation. This lack of significance may be attributed to the small number of cases with varying differentiation due to case distribution within the groups, or it may indicate that non-pure differentiation does not significantly impact the EMT and prognosis. Therefore, further studies with larger sample sizes of cases with concomitant differentiation are warranted.

Many studies have reported a highly significant positive correlation between the loss of membranous or cytoplasmic E-cadherin expression and vimentin expression [23,27,32]. However, a few studies have not found a significant relationship between E-cadherin and vimentin [30]. In our study, a positive correlation was observed between the loss of E-cadherin expression or cytoplasmic E-cadherin reaction and vimentin expression. When evaluating the effects of both antibodies in the context of the EMT, the results of most studies, including ours, suggest a complementary relationship between E-cadherin and vimentin.

## 5. Conclusions

In our study, we evaluated E-cadherin and vimentin expression in invasive areas to determine the EMT and then looked at galectin-3 expression in these areas. Most studies in the literature report that galectin-3 expression is increased in invasive tumor cells. However, in our study, we found that galectin-3 expression did not always increase in invasive tumor areas. In other words, we could not find a relationship between galectin-3, which is known to have invasion and migration functions, and the EMT. No statistically significant correlations were found between the expression of E-cadherin and vimentin in tumor budding areas and the invasive component and galectin-3 immunoreactivity in bladder urothelial carcinoma. This indicates that E-cadherin and vimentin expression is not associated with galectin-3 in this context. While the expression patterns of E-cadherin and vimentin in invasive areas support the concept of the EMT, the role and expression pattern of galectin-3 appear to be more complex compared to the other two antibodies. Our findings indicate that galectin-3 is influential and increases its expression during progression to high-grade tumors. Notably, there is a marked decrease in galectin-3 expression from the T1 to the T2 stage. Given these observations, further research is needed to better understand the relationship between galectin-3 and the EMT process in bladder urothelial carcinoma.

## Figures and Tables

**Table 1:** Staining intensity scores used for E-cadherin, vimentin, and galectin-3.

	Staining Intensity	Score
E-cadherin	Strong membranous staining	0
	Weak membranous staining or cytoplasmic staining	1
	Loss of staining	2
Vimentin and galectin-3	No staining	0
	Weak cytoplasmic staining	1
	Strong cytoplasmic staining	2

**Table 2:** Staining extensity scores used for E-cadherin, vimentin, and galectin-3.

	Staining Extensity	Score
E-cadherin	No loss of staining in invasive tumor cells	0
	Loss of staining in 1–10% of invasive tumor cells	1
	Loss of staining in 11–49% of invasive tumor cells	2
	Loss of staining in more than 50% of invasive tumor cells	3
Vimentin and galectin-3	No staining of invasive tumor cells	0
	1–10% of invasive tumor cells have staining	1
	11–49% of invasive tumor cells have staining	2
	More than 50% of invasive tumor cells have staining	3

**Table 3:** Demographic and histopathologic characteristics of the cases.

	T1 Low Grade (*n*:24)	T1 High Grade (*n*:105)	T2 High Grade (*n*:94)	*p* Value
Gender				0.77
Female	2 (8.3%)	11 (10.5%)	7 (7.4%)	
Male	22 (91.7%)	94 (89.5%)	87 (92.6%)	
Age (mean)	66.6	70.3	69.3	0.142
Divergent differentiation				<0.001
Present	-	12 (11.4%)	47 (50%)	
Lymphovascular invasion				0.04
Present	-	27 (25.7%)	58 (61.7%)	
Carcinoma in situ				<0.001
Present	-	20 (19%)	22 (23.4%)	

**Table 4:** Distribution of E-cadherin, vimentin, and galectin-3 staining intensity score between groups.

	E-Cadherin Staining Intensity Score			Vimentin Staining Intensity Score			Galectin-3 Staining Intensity Score		
	0 *n* (%)	1 *n* (%)	2 *n* (%)	0 *n* (%)	1 *n* (%)	2 *n* (%)	0 *n* (%)	1 *n* (%)	2 *n* (%)
T1 low grade (*n*:24)	19 (79)	5 (21)	-	24 (100)	-	-	11 (46)	12 (50)	1 (4)
T1 high grade (*n*:105)	65 (62)	27 (26)	13 (12)	87 (83)	8 (7)	10 (10)	34 (32)	38 (37)	33 (31)
T2 high grade (*n*:94)	12 (13)	67 (71)	15 (16)	67 (71)	13 (14)	14 (15)	51 (54)	33 (35)	10 (11)
*p* value	<0.001			0.023			0.012		

**Table 5:** Distribution of E-cadherin, vimentin, and galectin-3 staining extensity score between groups.

	E-Cadherin Staining Extensity Score				Vimentin Staining Extensity Score				Galectin-3 Staining Extensity Score			
	0 *n* (%)	1 *n* (%)	2 *n* (%)	3 *n* (%)	0 *n* (%)	1 *n* (%)	2 *n* (%)	3 *n* (%)	0 *n* (%)	1 *n* (%)	2 *n* (%)	3 *n* (%)
T1 low grade (*n*:24)	19 (79)	4 (17)	1 (4)	-	24 (100)	-	-	-	11 (46)	9 (37)	3 (13)	1 (4)
T1 high grade (*n*:105)	65 (62)	11 (10)	26 (25)	3 (3)	87 (83)	11 (10)	7 (7)	-	34 (32)	22 (21)	29 (28)	20 (19)
T2 high grade (*n*:94)	12 (13)	9 (10)	66 (70)	7 (7)	67 (71)	9 (10)	14 (15)	4 (4)	67 (71)	9 (10)	14 (15)	4 (4)
*p* value	<0.001				0.13				0.014			

## Data Availability

The original contributions presented in this study are included in the article; further inquiries can be directed to the corresponding author.
